# Ablation and laparoscopic adrenalectomy: Balancing efficacy and safety in the treatment of benign adrenal gland tumors: A systematic review and meta-analysis

**DOI:** 10.1016/j.heliyon.2024.e37868

**Published:** 2024-09-12

**Authors:** Benjamin Skribek, Anett Szabó, Júlia Ács, Péter Hegyi, Péter Mátrai, Péter Nyirády, Nándor Ács, Attila Majoros, Pál Ákos Deák

**Affiliations:** a Department of Interventional Radiology, Semmelweis University, Budapest, Hungary; b Centre for Translational Medicine, Semmelweis University, Budapest, Hungary; c Department of Urology, Semmelweis University, Budapest, Hungary; d Institute for Translational Medicine, Medical School, University of Pécs, Pécs, Hungary; e Institute of Pancreatic Diseases, Semmelweis University, Budapest, Hungary; f Department of Obstetrics and Gynecology, Semmelweis University, Budapest, Hungary

## Abstract

**Background:**

Aldosterone-producing adenomas cause hypertension in 5–10 % of cases. Laparoscopic adrenalectomy is the gold standard treatment for early-stage adrenal gland tumors, but minimally invasive procedures, such as ablative techniques can also be applied. Therefore, we aimed to compare laparoscopic adrenalectomy and ablation techniques in terms of efficacy and safety in the treatment of benign adrenal gland tumors.

**Materials and methods:**

We conducted a systematic search in five databases and included studies comparing the efficacy and safety of ablation techniques and laparoscopic adrenalectomy. We calculated odds ratios (ORs) for eligible studies with binary outcomes, and mean differences (MD) with 95 % confidence intervals (CI) for continuous outcomes.

**Results:**

Five studies focusing on aldosterone-producing adenomas were included in our review. A total of 119 patients at 14 centers underwent ablation, and 161 patients had laparoscopic adrenalectomy. The complication rates (OR: 0.98, CI: 0.35–2.69) were similar in both groups, but among complications, hypertensive crisis (OR: 8.13; CI: 1.14–58.11) was more frequent in the ablative group, and even the success rate of interventions - the resolution of hypertension (OR: 0.30, CI: 0.16–0.56) - was lower in this group. On the other hand, the advantage of ablation was shorter intervention time (MD: 75.64 min; CI: 6.33–144.95), shorter hospital stay (MD: 1.6 days; CI: 0.88–2.31), and less perioperative blood loss (MD: 43.55 ml; CI: 12.07–75.04) compared to laparoscopy.

**Conclusion:**

Laparoscopic adrenalectomy is still the best therapeutic approach, but ablation can be an appropriate alternative option for the treatment of aldosterone-producing adrenal gland tumors.

## 1 Introduction

The prevalence of adrenal tumors in the adult population ranges from 0.2 % to 3.2 % and tends to increase with age [1]. Histologically, these tumors are mainly adenomas that can transform into hormone-producing adrenal masses with higher malignant potential [[2], [3], [4]] Aldosterone-producing adenomas (APA) lead to primary hyperaldosteronism (PH), with symptoms of high blood pressure and low levels of potassium in the blood. APA causes PH in 33 % of the cases [5]. Studies have shown that primary hyperaldosteronism is a relatively rare condition but accounts for 5–10 % of all cases of hypertension, with possible complications of stroke, myocardial infarction, cardiac arrhythmias, kidney failure, and so on [[6], [7], [8]]. The diagnosis of APA is based on adrenal vein sampling (AVS) from both adrenal veins and imaging tests, such as computed tomography (CT) or magnetic resonance imaging (MRI) scans [7,8].

Currently, laparoscopic adrenalectomy (LA) is the gold standard treatment for primary hyperaldosteronism associated with APA. However, limiting factors, such as obesity, previous abdominal surgeries, coagulopathies, and cardiopulmonary diseases, restrict the use of LA; thus, alternative therapeutic options should be sought [9]. Additionally, during an adrenalectomy, there is a risk of injury to various anatomical structures, such as the colon, pancreas, spleen, and diaphragm, that surround the adrenal glands [10]. Only a handful of studies investigated alternatives to LA and compared them in terms of complication and success rates.

Alternatives of LA include ultrasound- and percutaneous CT-guided cryotherapy, microwave- (MWA) or radiofrequency ablation (RFA) procedures, catheter-based ethanol ablation, and adrenal artery embolization techniques, all of which are exclusively for the management of benign adrenal gland tumors [[11], [12], [13]]. Microwave ablation is the least invasive therapeutic option; however, radiofrequency ablation (RFA) is the most successful and commonly performed ablation technique [14,15]. Hormone-producing adrenal gland tumors can be cured via ablation in selected individuals, but the risk of a hypertensive crisis is significantly higher than for LA [[16], [17], [18]].

Therefore, we aimed to summarize the comparison of laparoscopic adrenalectomy and minimally invasive procedures in terms of complications and biochemical success in the treatment of benign adrenal tumors. We also sought to assess if interventional approaches, such as ablation techniques, could replace laparoscopy as the preferred gold standard of care.

## 2 Materials and methods

We followed the recommendations of the Cochrane Handbook, Preferred Reporting Items for Systematic Reviews and Meta-Analyses (PRISMA) and the second version of AMSTAR (Assessing the methodological quality of systematic reviews) statement during the selection and extraction phases (see Supplementary Table 1) [[19], [20], [21]]. Our study protocol was registered on PROSPERO before the start date with the registration number CRD42022367148.

### 2.1 Literature search

Five databases, MEDLINE via PubMed, Central, Scopus, Web of Science, and Embase were searched for relevant articles. The search date was November 5, 2022, and the last update was on July 25, 2024; the main domains of our search key were adrenal tumors, laparoscopy, and minimally invasive interventions (Supplementary Table 2).

No filters or other restrictions were used.

### 2.2 Eligibility criteria

We formulated our clinical question using the PICO (Population, Intervention, Comparison, Outcome) framework. The identified studies were selected according to the following criteria: (P) patients who underwent LA or an interventional radiological procedure to treat early-stage adrenal gland tumors (I, C). Possible ablative techniques included cryoablation, radiofrequency ablation, microwave ablation, chemoablation, intravascular embolization, high-intensity focused ultrasound (HIFU), laser therapy, silicone gel therapy, or irreversible electroporation, which were compared to laparoscopic adrenalectomy; (O) in terms of outcomes: complication rate, which was calculated based on mortality, major and minor morbidity factors; the proportion of hypertensive crisis; resolution of hypertension, described as achieving a normalized aldosterone-to-renin ratio (ARR) and no longer requiring additional antihypertensive medications following interventions; perioperative blood loss; operation time; length of hospital stay; and postoperative pain treatment. The study design included cohort studies, case-control studies, and randomized controlled trials. No language restrictions were applied to the selection process.

Studies were excluded if they were (a) reviews, meta-analyses, systematic reviews, case reports, and case series; (b) non-comparative studies; (c) preclinical or animal studies; (d) research on patients with advanced tumors or metastases. Data from conference abstracts and papers for which the full text could not be retrieved were also excluded.

### 2.3 Study selection process

**Fig. 1:**
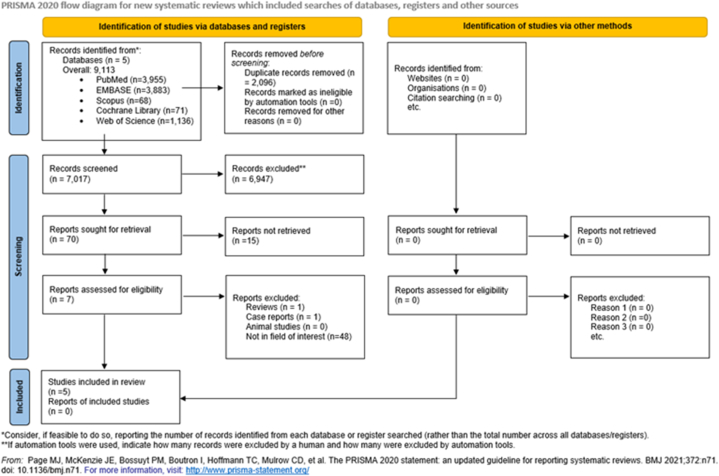
PRISMA 2020 flowchart showing the study selection process.

Endnote v9.0 (Clarivate Analytics, Philadelphia, PA, USA) reference manager software and Ryyan (Rayyan Systems Inc., Cambridge, MA 02142, USA) were used to select studies. After the automatic and manual removal of duplicate records, two co-investigators (BS and JÁ) separately evaluated the eligibility of articles by first author, title, and abstract, then the remaining articles by full text. Discrepancies were resolved by a third investigator (AS). To check reliability, Cohen's kappa coefficient (κ) was calculated after each step [22]. The complete study selection process was illustrated in the PRISMA flowchart (Fig. 1).

### 2.4 Data extraction

Two authors (BS and JÁ) independently used a standardized data extraction table to extract data from eligible articles. The extracted data involved (a) general information on the article: name of the first author, year of publication, study design, study region, type of ablation procedure, and study period; (b) characteristics of the population: age, sex, tumor diameter, and location, highest systolic and diastolic blood pressure; (c) primary outcome parameters: complications, hypertensive crisis, biochemical success in terms of hypertension resolution, and secondary outcome parameters: perioperative blood loss, operation time, length of hospital stay and postoperative pain treatment.

The preferred data format for operation time, length of hospital stay, and perioperative blood loss was the mean with standard deviation (SD). The mean and standard deviation of blood loss were estimated from the median and quartile values based on the work by Sun et al. [23]. Furthermore, the data on hospital stays from Cano et al. were calculated from the median, minimum, and maximum ranges for mean and SD [16].

### 2.5 Risk of bias (quality) assessment

Two independent review authors (BS and JÁ) used the Quality in Prognosis Studies (QUIPS) tool to assess the risk of bias as recommended by the Cochrane Handbook [19,24]. Categories of risk assessment were pre-defined for each domain (Supplementary Appendix). Disagreements were resolved by a third review author (AM).

### 2.6 Statistical analysis

The statistical analyses were performed using the R software (R Core Team 2020, version 4.0.3), and the meta (version 6.1-0) package was used for calculations and plots [25].

For continuous outcomes, the mean difference (MD) was calculated as the effect size measure with a 95 % confidence interval (CI). To calculate the MD and its standard deviation, the extracted values were the sample size, the mean, and the standard deviation for both groups. In the rare cases where a study did not report these, the mean was estimated using the method proposed by Luo et al., and the standard deviation was calculated using the method proposed by Shi et al. based on the median, quartile, minimum, and maximum values [26,27]. When such a calculation was needed, we indicated it on the forest plot.

The odds ratio (OR) with 95 % CI was used to measure the effect of binary outcomes. The OR was calculated by subtracting the total number of patients in each group and the total number of patients with the event of interest from each study. For the summary OR estimate, we used the Mantel-Haenszel method (without continuity correction) with the Paule-Mandel estimator for the between-study variation τ following the recommendation of Harrer [28].

Raw data from the selected studies were summarized using the random-effects model. We used the Hartung-Knapp adjustment for each analysis to avoid false positive conclusions [29]. Where applicable, we reported the 95 % summary prediction interval (PI), following the recommendation of IntHout [30].

Cochrane's Q test was evaluated to assess statistical heterogeneity, while the I2 index was utilized to quantify the extent of heterogeneity between studies. Additionally, funnel plots were employed to report and visually represent any potential publication bias effectively. We performed a sensitivity analysis using the "Leave one out" method.

We used forest plots to summarize the results graphically. Statistical significance was defined as p-value <0.05 for all outcomes.

No subgroup analysis was performed.

## 3 Results

### 3.1 Search and selection

A total of 7017 articles were found, of which five studies were eligible for our meta-analysis and systematic review (Fig. 1).

### 3.2 Basic characteristics of included studies

The five articles used were all retrospective cohort studies. The baseline characteristics of these studies are detailed in Table 1. All studies focused on adrenal aldosterone-producing adenomas. Altogether, 119 patients were treated with adrenal ablation, and 161 underwent laparoscopic adrenalectomy in 14 centers in Europe, Asia, and the USA. CT or MRI imaging and adrenal venous sampling were performed in most cases prior to the diagnosis of APA. In four studies, percutaneous CT-guided radiofrequency ablation was the ablative procedure of choice, and catheter-based ethanol ablation was used only in one case. Data on treatment are detailed in Supplementary Table 5, and the eligibility criteria for each study are summarized in Supplementary Table 4.

**Table 1:** Basic characteristics of included studies.

Author (year)	Study site	Study design	Study period	Study participation	Age (year)^a^ Ablation group	Age (year)^a^ LA group	Sex (female% of total)	Follow-up period (months)^a^
Liu et al. (2016) [15]	China	Retrospective	2004–2012	63	52.2 ± 10.4	50.7 ± 10.3	55.6	68.4 (22.8–127.2)
Yang et al. (2016) [32]	Taiwan	Retrospective	2009–2013	25	54 (29–74)	45 (20–69)	56	ND (3–6)
Sarwar et al. (2016) [17]	USA	Retrospective	2008–2013	44	51 ± 11	50 ± 11	47.7	11.4 ± 12.8
Cano-Valderrama et al. (2021) [16]	Spain	Retrospective	2007–2019	34	54.3 (49.1–59.5)	55.5 (50.3–60.8)	35.3	46.2(3.4–147.5)
Sun et al. (2022) [23]	China	Retrospective	2016–2019	112	45 (36–52)	43 (37–50)	50.1	6 (ND)

LA: Laparoscopic adrenalectomy; ND: not defined; USA: United States of America.

a Parameters represented as mean with standard deviation, or median with range (minimum and maximum).

### 3.3 Resolution of hypertension

**Fig. 2:**
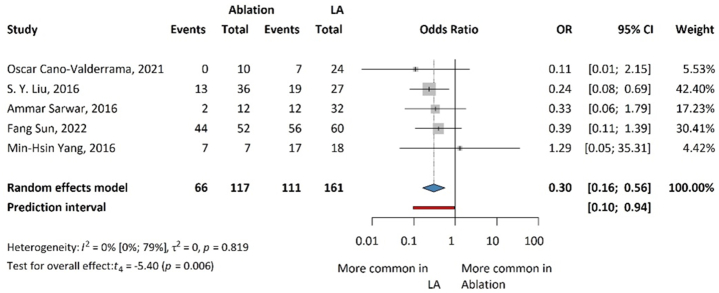
Forest plot summarizing the odds ratios (OR) of resolution of hypertension associated with ablation and laparoscopic adrenalectomy for aldosterone-producing adrenal tumors.

Results for the resolution of hypertension are presented in Fig. 2. Compared with laparoscopic adrenalectomy and ablation in resolving hypertension, i.e., biochemically measured clinical success, LA was significantly more effective than ablation (OR: 0.30; 95 % CI: 0.16–0.56; p = 0.006).

### 3.4 Complications

**Fig. 3:**
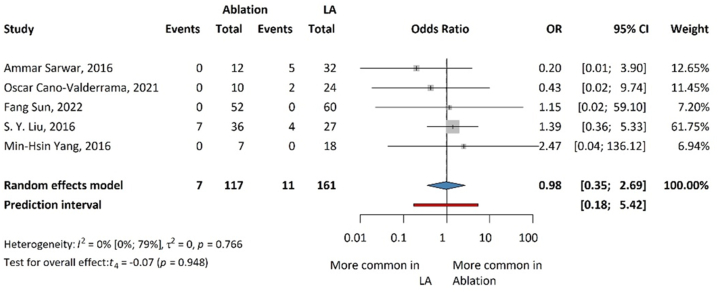
Forest plot summarizing the odds ratios (OR) of complications associated with ablation and laparoscopic adrenalectomy for aldosterone-producing adrenal tumors.

The results for complications are summarized in Fig. 3. The complication rate was determined by considering mortality and major and minor complications, excluding hypertensive crises. No clinically relevant differences were found between the ablation procedures and laparoscopic adrenalectomy (OR: 0.98; 95 % CI: 0.35–2.69; p = 0.948). However, our results are not statistically significant.

### 3.5 Hypertensive crisis

**Fig. 4:**
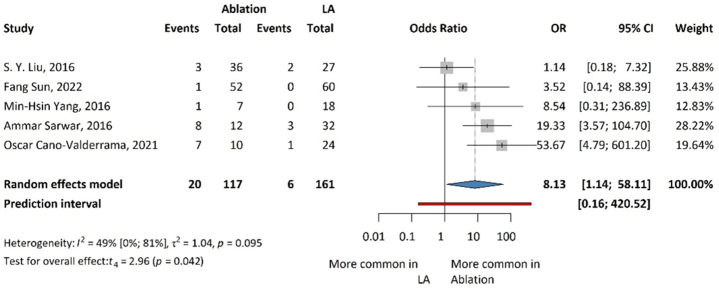
Forest plot summarizing the odds ratios (OR) of hypertensive crisis associated with ablation and laparoscopic adrenalectomy for aldosterone-producing adrenal tumors.

Results for the hypertensive crisis are presented in Fig. 4, revealing significant differences between ablation and laparoscopic adrenalectomy (OR: 8.13; 95 % CI: 1.14–58.11; p = 0.042) with the results in favor of surgery. Ablation has a statistically significant higher probability of being associated with hypertensive crisis.

### 3.6 Operation time

Operation times are summarized in Supplementary Fig. 1. Data on operation time were available in four studies. For ablation procedures, intervention times were significantly shorter than for LA (MD: 75.64 min; 95 % CI: 6.33–144.95; p = 0.040), with intervention times varying from an average of 12–158 min.

### 3.7 Length of hospital stay

Data on length of hospital stay (LOS) were reported in four studies and are summarized in Supplementary Fig. 2. Postoperative hospitalization time was significantly shorter in the ablation group than in the laparoscopic adrenalectomy group (MD: 1.6 days; 95 % CI: 0.88–2.31; p = 0.006).

### 3.8 Perioperative blood loss, postoperative pain treatment

The reviewed studies indicated a higher perioperative blood loss with laparoscopic adrenalectomy. Sun et al.'s research showed less blood loss with ablation (3.41 ± 3.05 ml) compared to laparoscopy (48.26 ± 60.70 ml), and the study by Sarwar et al. also favored ablation procedures (1.20 ± 3.00 ml versus 40.00 ± 85.00 ml). In addition, both Liu et al. and Sarwar et al. observed less postoperative pain in the ablation group than in LA. Results are summarized in Supplementary Figs. 3 and 4.

### 3.9 Risk of bias assessment

The results of the risk of bias assessment are presented in Supplementary Table 6. The risk of bias for study participation was mainly low, whereas study attrition was not applicable to any of the studies due to their retrospective design. The risk of bias was mostly moderate for prognostic factors and outcome measurement. Next, we evaluated study confounding, which was moderate in all included articles. Finally, a low risk of bias was attributed to statistical analyses. Sensitivity analysis was performed using the Leave-One-Out test, demonstrating our analysis's robustness as it showed minimal sensitivity.

### 3.10 Publication bias and heterogeneity

Egger's test could not be performed due to the low number of articles.

Significant heterogeneity was found for operation time (I2 = 91 %, 95 % CI: 81 %; 96 %) and postoperative pain treatment (I2 = 84 %, 95 % CI: 36 %; 96 %), and heterogeneity was moderate for hypertensive crisis (I2 = 49 %, 95 % CI: 0 %; 81 %) and hospital stay (I2 = 37 %, 95 % CI:0 %; 78 %).

## 4 Discussion

This meta-analysis identified five studies that investigated the differences between ablation and laparoscopic adrenalectomy for benign adrenal gland tumors in terms of safety and efficacy. LA is considered to be a more invasive procedure requiring more time to be performed and resulting in a longer recovery time compared to adrenal ablation in the treatment of aldosterone-producing adenomas; however, LA has been shown to be more effective in the treatment of hypertension. On the other hand, in terms of complications, the disadvantage of ablation is that it is more likely to result in a hypertensive crisis, a potentially life-threatening condition. Therefore, by weighing the advantages and disadvantages of the two procedures, ablation should be recommended to patients unsuitable for surgery, such as those with extreme obesity, previous abdominal surgeries, coagulopathies, and cardiopulmonary diseases. Ablation may also be an option for those favoring a less invasive procedure. Based on all the results, laparoscopic adrenalectomy remains the gold standard therapy for the treatment of hormone-producing adrenal tumors, as it is both more effective and safer than adrenal ablation.

Two meta-analyses were previously published on this topic, comparing radiofrequency ablation with laparoscopic adrenalectomy regarding efficacy and safety. The first meta-analysis published by Chen et al. (2021) included five comparative studies, partially overlapping with some studies we also found eligible [31]. In three studies, clinical success was defined as the biochemical resolution of primary hyperaldosteronism and normalization of the aldosterone-to-renin ratio [15,32,33]. The remaining two studies defined it as the resolution of hypertension without additional antihypertensive medications after interventions [17,34]. Overall, the odds ratio was 0.55 (95 % CI: 0.22–1.37) in favor of laparoscopy. In our study, the resolution of hypertension was the main element of clinical effectiveness, with an odds ratio of 0.30 (95 % CI: 0.16–0.56). In the study by Chen, complications were divided into three groups, which were minor (OR: 1.19; 95 % CI: 0.50, 2.84) and major complications (OR: 0.45; 95 % CI:0.08–2.39) or hypertensive crisis (OR: 5.53; 95 % CI: 0.77–39.79). Their results were consistent with ours regarding secondary results – operation times, length of hospital stay, and perioperative blood loss. One of the major strengths of the study by Chen is that all included articles are comparative, which analyzed several outcomes, such as changes in serum potassium levels, decrease of diastolic and systolic blood pressure, and decrease in aldosterone-to-renin ratio. Despite the aforementioned advantages, the meta-analysis by Chen had certain limitations, most notably that it used duplicate patient series from two articles [15,33]. Additionally, the included studies used different surgical procedures – Yang et al. used retroperitoneoscopic-guided cool-tip radiofrequency ablation, whereas the other studies used CT-guided RFA [34]. In 2021, Guo et al. also published a meta-analysis comparing ablative techniques with laparoscopic adrenalectomy in the treatment of adrenal gland tumors [35]. An odds ratio of 0.72 (95 % CI:0.22–2.39) was established for clinical success, favoring surgery over radiofrequency ablation in treating aldosterone-producing adenoma-related hypertension. Generally, complications were more frequent in the laparoscopic group (OR: 0.67; 95 % CI: 0.27–1.68) than in the ablative group, but the most severe complication - hypertensive crisis - was more than three times more likely to occur (OR: 3.16; 95 % CI: 0.36–27.94) during ablation. A limitation of this study was the inclusion of a comparative study by Yang et al. in which the ablation was guided by laparoscopy [34].

Laparoscopic adrenalectomy via the transabdominal lateral approach is the gold standard for treating adrenal diseases, including tumors up to 12–15 cm, while the posterior retroperitoneal approach is an excellent minimally invasive alternative for benign and medium-sized tumors (<6 cm) [36]. Laparoscopic transperitoneal adrenalectomy involves entering the abdominal cavity through incisions in the front, providing a more expansive operative space and better visualization, making it ideal for larger or complex tumors. Retroperitoneoscopic adrenalectomy, accessed through the back, avoids traversing abdominal organs, reducing the risk of complications but offers a more confined working space. Transabdominal laparoscopy is preferred for larger, and more complex tumors, while the retroperitoneoscopic approach suits smaller tumors or patients with prior abdominal surgeries. The choice between these approaches depends on tumor size, location, patient anatomy, and the surgeon's expertise [37]. Laparoscopic and retroperitoneoscopic procedures promote earlier mobilization and recovery, reduce the risk of pulmonary infections and thromboembolic complications, and have lower morbidity and mortality rates compared to traditional surgery. In a study by Conzo et al., 126 patients underwent laparoscopic adrenalectomy at a single center. Of these, 84 patients had functioning tumors, including 27 with pheochromocytoma, 29 with Conn's disease, and 28 with Cushing's syndrome. Thirty-eight patients were diagnosed with incidentaloma, and one patient had myelolipoma. Three patients had adrenal metastasis from breast and renal cancers. After laparoscopy, no malignant primary tumors were found in the final pathology. There were 16 intraoperative hypertensive crises reported, but no major postoperative complications occurred [38].

In recent decades, only a few prospective and retrospective studies have been published on the effectiveness and safety of radiofrequency ablation. One of these articles included 13 patients, 10 of whom had aldosterone-producing adenomas, one of the other three had a pheochromocytoma, another patient had a testosterone-secreting tumor, and the last one had a cortisol-secreting adenoma [39]. RFA was successfully performed in all cases as it resulted in the resolution of hypertension, virilization, Cushing syndrome, and all the other symptoms, respectively. Complications during the procedures included a small pneumothorax, a limited hemothorax, and two cases of hypertensive urgencies, but the patients were discharged within 24 h of the intervention without further complications.

The most common and dreaded complication of radiofrequency ablation is a hypertensive crisis; therefore, extra precautions must be taken. To prevent this complication, patients are usually prescribed alpha- and beta-blocking drugs before the intervention for both functional and non-functional adrenal tumors [40]. This medication helps to avoid hypertensive urgency and reduce the risk of complications during the procedure. Non-hormonally active adrenal metastases are found to be significantly associated with hypertensive crisis if the maximum tumor diameter is less than 4.5 cm, and normal adrenal tissue can be seen on CT or MR imaging before the procedure [40]. In a previous meta-analysis, Liang et al. reported a prevalence of 19.1 % for hypertensive crisis during ablation [41]. According to the article by Pan et al., 21.9 % of 959 patients who had adrenal metastases experienced an episode of hypertensive urgency during interventional procedures [42]. In this study, radiofrequency ablation, microwave ablation, cryoablation, and ethanol ablation were also included.

A field strongly connected to benign adrenal gland tumors is the treatment of malignant adrenal masses, for which minimally invasive interventions are increasingly available as well. Based on the results of Liu et al., image-guided radiofrequency ablation proved to be a successful procedure with 19 % of local tumor recurrence, 82 % of 1-year overall survival (OS), and a 7 % pooled ratio of hypertensive crisis in a total of 351 patients treated [43]. In a further study by Pan et al., the pooled 1-year local control rate, defined as a tumor volume reduced or at least equivalent to the initial tumor volume, was 80 %, and the pooled 1-year overall survival rate was 77 % [42]. In a sample of 959 patients related to this research, 21.9 % of patients experienced intraprocedural hypertensive urgency. In the article by Kelly C. Harper et al., 57 adrenal metastases from renal cell carcinoma were treated with cryoablation in 46 patients [44]. The ablation aim was curative for 39 out of 57 tumors (72 %), with local tumor control being the goal for the remaining cases. The recurrence-free survival rates at 1, 3, and 5 years were 100 %, 89 %, and 89 %, respectively. According to the study by Botsa et al., local recurrence occurred in 22.8 % of the 35 patients treated with RFA and 19.4 % of the 36 patients treated with MWA [45]. Zhou et al. found that the recurrence rate among 33 patients treated with RFA was 24.2 %, with an average recurrence-free period of 27.4 months [46]. These results confirm that image-guided percutaneous ablation can be an effective therapeutic option with a moderate safety profile for treating adrenal metastases.

### 4.1 Strengths and limitations

As for the strengths of our analysis, we followed our protocol, which was registered in advance. All included articles compared ablative procedures with laparoscopic adrenalectomy within the same article, and all had similar definitions of outcomes. Data were available for radiofrequency and ethanol ablations as well. Our analysis focuses exclusively on percutaneous ablation and does not include duplicate patient cases or data from retroperitoneoscopic-guided radiofrequency ablation. Sensitivity analysis was performed using the Leave-One-Out test, demonstrating our analysis's robustness as it showed minimal sensitivity.

Our study had some limitations. First, only retrospective studies were available, with few patients. The included studies were heterogeneous in design, data collection methods, and eligibility criteria. Data extraction was limited for secondary outcomes due to a lack of reporting. In addition, considerable heterogeneity was found, mainly for intervention times and postoperative pain treatments. Furthermore, the studies included in the analysis predominantly exhibit a moderate level of risk when assessed for bias. Lastly, our study contains data solely on aldosterone-producing adrenal adenomas.

## 5 Conclusion

Minimally invasive procedures are more likely to lead to intraoperative hypertensive crises and result in lower biochemical effectiveness, but on the other hand, they are associated with shorter operation time and hospital stay, less intraoperative blood loss, and less postoperative pain compared to laparoscopic adrenalectomy. Our results suggest that LA remains the gold standard therapy for the treatment of hormone-producing adrenal tumors.

### Implications for practice and research

Implementing scientific findings is pivotal in delivering benefits to the community [47,48]. In clinical practice, laparoscopic adrenalectomy is the most appropriate procedure for the treatment of aldosterone-producing tumors, but if LA is not feasible, ablation procedures may be considered as alternative therapeutic options. However, more comparative studies, mainly randomized controlled trials, are needed to evaluate the efficacy and safety of interventional procedures versus laparoscopic adrenalectomy.

## Funding

Funding was provided by 10.13039/501100002332Semmelweis University. Sponsors had no role in the design, data collection, analysis, interpretation, and manuscript preparation.

## Ethical approval

No ethical approval was required for this systematic review with meta-analysis, as all data had already been published in peer-reviewed journals. No patients were involved in the design, conduct, or interpretation of our study.

## Data availability

The datasets used in this study can be found in the full-text articles included in the systematic review and meta-analysis.

Has data associated with your study been deposited into a publicly available repository? No.

Data included in article/supp. material/referenced in article.

## CRediT authorship contribution statement

**Benjamin Skribek:** Writing – original draft, Project administration, Methodology, Formal analysis, Conceptualization. **Anett Szabó:** Writing – review & editing, Visualization, Formal analysis, Conceptualization. **Júlia Ács:** Writing – review & editing, Visualization, Formal analysis, Conceptualization. **Péter Hegyi:** Writing – review & editing, Funding acquisition, Conceptualization. **Péter Mátrai:** Writing – review & editing, Data curation, Conceptualization. **Péter Nyirády:** Writing – review & editing, Conceptualization. **Nándor Ács:** Writing – review & editing, Conceptualization. **Attila Majoros:** Writing – review & editing, Conceptualization. **Pál Ákos Deák:** Writing – original draft, Supervision, Conceptualization.

## Declaration of competing interest

The authors declare that they have no known competing financial interests or personal relationships that could have appeared to influence the work reported in this paper.

## Abbreviations

AMSTAR: Assessing the methodological quality of systematic reviews
APA: Aldosterone-producing adenoma
AVS: Adrenal vein sampling
ARR: Aldosterone-to-renin ratio
CI: Confidence interval
CT: Computed tomography
HIFU: High-intensity focused ultrasound
LA: Laparoscopic adrenalectomy
LOS: Lengths of hospital stay
MD: Mean difference
MRI: Magnetic resonance imaging
MWA: Microwave ablation
OR: Odds ratio
OS: Overall survival
PH: Primary hyperaldosteronism
PICO: Population, intervention, comparison, outcome
PI: Prediction interval
PRISMA: Preferred Reporting Items for Systematic Reviews and Meta-Analyses
RFA: Radiofrequency ablation
SD: Standard deviation
QUIPS: Quality in Prognosis Studies
