# Supplementary material for: Ablation and laparoscopic adrenalectomy: Balancing efficacy and safety in the treatment of benign adrenal gland tumors: A systematic review and meta-analysis
Source: Heliyon. 2024 Sep 12;10(19):e37868. doi: 10.1016/j.heliyon.2024.e37868 (PMC11462193; doi:10.1016/j.heliyon.2024.e37868)

**Supplementary Material**

**Title**

**Ablation and Laparoscopic Adrenalectomy: Balancing Efficacy and Safety in the Treatment of Adrenal Gland Tumors: A Systematic Review and Meta-analysis**

**TABLE OF CONTENT**

**Supplementary Appendix 1.** Risk of bias assessment methodology

**Supplementary Table 1.** PRISMA 2020 checklist

**Supplementary Table 2.** The list of keywords for the systematic search

**Supplementary Table 3.** Exclusion rationale for studies eliminated during full-text screening

**Supplementary Table 4.** Eligibility criteria of each included article

**Supplementary Table 5.** Baseline diagnostic and treatment characteristics of included studies

**Supplementary Table 6.** Risk of bias assessment using the QUIPS tool

**Supplementary Figure 1.:** Summary forest plot of operation time (minutes)

**Supplementary Figure 2.:** Summary forest plot of length of hospital stay (days)

**Supplementary Figure 3.:** Summary forest plot of perioperative blood loss (ml)

**Supplementary Figure 4.:** Summary forest plot of postoperative pain treatment

**Supplementary Appendix 1.** Risk of bias assessment methodology

**Study participation measurement:** (1) low risk of bias was attributed if authors adequately described the source population, including methods to identify patients and eligibility criteria. The description of the period and place of recruitment were also added to low risk of bias; (2) moderate risk of bias was attributed if a part of the above-listed descriptions were missing; (3) high risk of bias was attributed if baseline characteristics, eligibility criteria, time and place of recruitment were not described.

**Study attrition assessment** was not applicable (NA) for either of the included studies due to their retrospective nature.

**Prognostic factor measurement:** (1) low risk of bias was attributed if adrenal tumors were diagnosed with the most up-to-date methods (CT/MRI imaging and AVS); (2) moderate risk of bias was attributed if the diagnosis was acquired by inferior methods, such as laboratory tests, but still, it was mentioned; (3) high risk of bias was attributed if the presence of an adrenal tumor was only stated without any indication of measurement.

**Outcome measurement:** (1) low risk of bias was attributed if complications were adequately detailed and data on secondary outcomes were also included; (2) moderate risk of bias was attributed if secondary outcomes were somewhat lacking; (3) high risk of bias was attributed if secondary outcomes were not assessed.

**Study confounding measurement:** (1) low risk of bias was attributed if important potential confounders were described and accounted for in the analysis, preferably in a table; (2) moderate risk of bias was attributed if some of the important confounders were described (at least 1-2 other possible risk factors); (3) high risk of bias was attributed if studies did not provide data on confounding factors.

**Statistical analysis measurement**: (1) low risk of bias was attributed if data were presented as mean and standard deviation; (2) moderate risk of bias was attributed if standard error or median with minimum and maximum values or quartiles were given; (3) high risk of bias was attributed if the data were incompletely presented.

**Supplementary Table 1.** PRISMA 2020 checklist

| 1. **Section and topic** | **Item #** | **Checklist item** | **Location where item is reported** |  |
| --- | --- | --- | --- | --- |
| **Title** | | | | |
| Title | 1 | Identify the report as a systematic review. | 1 |  |
| **Abstract** | | | | |
| Abstract | 2 | See the PRISMA 2020 for Abstracts checklist (table 2). | 2 Supplementary Table 1 |  |
| **Introduction** | | | | |
| Rationale | 3 | Describe the rationale for the review in the context of existing knowledge. | 3-4 |  |
| Objectives | 4 | Provide an explicit statement of the objective(s) or question(s) the review addresses. | 4 |  |
| **Methods** | | | | |
| Eligibility criteria | 5 | Specify the inclusion and exclusion criteria for the review and how studies were grouped for the syntheses. | 4-5 |  |
| Information sources | 6 | Specify all databases, registers, websites, organizations, reference lists and other sources searched or consulted to identify studies. Specify the date when each source was last searched or consulted. | 4 |  |
| Search strategy | 7 | Present the full search strategies for all databases, registers, and websites, including any filters and limits used. | 4 |  |
| Selection process | 8 | Specify the methods used to decide whether a study met the inclusion criteria of the review, including how many reviewers screened each record and each report retrieved, whether they worked independently, and if applicable, details of automation tools used in the process. | 5 |  |
| Data collection process | 9 | Specify the methods used to collect data from reports, including how many reviewers collected data from each report, whether they worked independently, any processes for obtaining or confirming data from study investigators, and if applicable, details of automation tools used in the process. | 6 |  |
| Data items | 10a | List and define all outcomes for which data were sought. Specify whether all results that were compatible with each outcome domain in each study were sought (e.g., for all measures, time points, analyses), and if not, the methods used to decide which results to collect. | 6 |  |
|  | 10b | List and define all other variables for which data were sought (e.g., participant and intervention characteristics, funding sources). Describe any assumptions made about any missing or unclear information. | 6 |  |
| Study risk of bias assessment | 11 | Specify the methods used to assess risk of bias in the included studies, including details of the tool(s) used, how many reviewers assessed each study and whether they worked independently, and if applicable, details of automation tools used in the process. | 6 |  |
| Effect measures | 12 | Specify for each outcome the effect measure(s) (e.g., risk ratio, mean difference) used in the synthesis or presentation of results. | 6-7 |  |
| Synthesis methods | 13a | Describe the processes used to decide which studies were eligible for each synthesis (e.g., tabulating the study intervention characteristics and comparing against the planned groups for each synthesis (item #5)). | 6-7 |  |
|  | 13b | Describe any methods required to prepare the data for presentation or synthesis, such as handling of missing summary statistics, or data conversions. | 6-7 |  |
|  | 13c | Describe any methods used to tabulate or visually display results of individual studies and syntheses. | 6-7 |  |
|  | 13d | Describe any methods used to synthesize results and provide a rationale for the choice(s). If meta-analysis was performed, describe the model(s), method(s) to identify the presence and extent of statistical heterogeneity, and software package(s) used. | 6-7 |  |
|  | 13e | Describe any methods used to explore possible causes of heterogeneity among study results (e.g. subgroup analysis, meta-regression). | 6-7 |  |
|  | 13f | Describe any sensitivity analyses conducted to assess robustness of the synthesized results. | 6-7 |  |
| Reporting bias assessment | 14 | Describe any methods used to assess risk of bias due to missing results in a synthesis (arising from reporting biases). | 7 |  |
| Certainty assessment | 15 | Describe any methods used to assess certainty (or confidence) in the body of evidence for an outcome. | 7 |  |
| **Results** | | | | |
| Study selection | 16a | Describe the results of the search and selection process, from the number of records identified in the search to the number of studies included in the review, ideally using a flow diagram (see fig 1). | 8  Figure 1 |  |
|  | 16b | Cite studies that might appear to meet the inclusion criteria, but which were excluded, and explain why they were excluded. | Supplementary Table 3 |  |
| Study characteristics | 17 | Cite each included study and present its characteristics. | Table 1  Supplementary  Table 4 |  |
| Risk of bias in studies | 18 | Present assessments of risk of bias for each included study. | Supplementary  Table 6 |  |
| Results of individual studies | 19 | For all outcomes, present, for each study: (a) summary statistics for each group (where appropriate) and (b) an effect estimate and its precision (e.g. confidence/credible interval), ideally using structured tables or plots. | Figures 2-4  Supplementary Figures 1-4 |  |
| Results of syntheses | 20a | For each synthesis, briefly summarize the characteristics and risk of bias among contributing studies. | 10  Table 1  Supplementary Table 5 |  |
|  | 20b | Present results of all statistical syntheses conducted. If meta-analysis was done, present for each the summary estimate and its precision (e.g. confidence/credible interval) and measures of statistical heterogeneity. If comparing groups, describe the direction of the effect. | 8-10  Figures 2-4  Supplementary Figures 1-4 |  |
|  | 20c | Present results of all investigations of possible causes of heterogeneity among study results. | 10 |  |
|  | 20d | Present results of all sensitivity analyses conducted to assess the robustness of the synthesized results. | 10 |  |
| Reporting biases | 21 | Present assessments of risk of bias due to missing results (arising from reporting biases) for each synthesis assessed. | 10 |  |
| Certainty of evidence | 22 | Present assessments of certainty (or confidence) in the body of evidence for each outcome assessed. | 8-10  Figures 2-4  Supplementary Figures 1-4 |  |
| **Discussion** | | | | |
| Discussion | 23a | Provide a general interpretation of the results in the context of other evidence. | 10-14 |  |
|  | 23b | Discuss any limitations of the evidence included in the review. | 13-15 |  |
|  | 23c | Discuss any limitations of the review processes used. | 13-15 |  |
|  | 23d | Discuss implications of the results for practice, policy, and future research. | 15 |  |
| **Other information** | | | | |
| Registration and protocol | 24a | Provide registration information for the review, including register name and registration number, or state that the review was not registered. | 4 |  |
|  | 24b | Indicate where the review protocol can be accessed, or state that a protocol was not prepared. | 4 |  |
|  | 24c | Describe and explain any amendments to information provided at registration or in the protocol. | NA |  |
| Support | 25 | Describe sources of financial or non-financial support for the review, and the role of the funders or sponsors in the review. | 17 |  |
| Competing interests | 26 | Declare any competing interests of review authors. | 17 |  |
| Availability of data, code, and other materials | 27 | Report which of the following are publicly available and where they can be found: template data collection forms; data extracted from included studies; data used for all analyses; analytic code; any other materials used in the review. | 17 |  |

**Supplementary Table 2.** The list of keywords for the systematic search

| **Databases** | **Search key** |
| --- | --- |
| Pubmed  EMBASE  Cochrane Library  Scopus  Web of Science | (non-invasive OR noninvasive OR silicone gel OR endovenous OR endovascular OR transvenous OR chemical OR percutan* OR ethanol OR catheter* OR high-intensity focused ultrasound OR high intensity focused ultrasound OR HIFU OR laser* OR IRE OR irreversible electroporation OR mechanic* OR embolization OR embolisation OR thermo* OR radiofrequency OR RFA OR microwave OR MWA OR ablation OR cryo*) **AND** (adrenal adenoma OR adrenal tumor* OR adrenal tumour OR adrenal gland* OR adrenal cancer* OR adrenal neoplasia OR adrenal neoplasm) **AND** (adrenalectomy OR surgery OR resection OR laparosc* OR endoscop* OR invasive) |

*****We used the same keywords in each database.

**Supplementary Table 3.** Exclusion rationale for studies eliminated during full-text screening

| **Author (year)** | **DOI** | **Reason for exclusion** |
| --- | --- | --- |
| A. Abbas (2013) | 10.1016/j.crad.2012.06.137 | Not comparative study |
| Z. Al-Hilli (2018) | 10.1007/978-3-319-92860-9_31 | Review study |
| Almeida (2013) | NA | Conference abstract |
| S. P. Balasubramanian (2014) | 10.1016/j.mpsur.2014.07.003 | Not comparative study |
| D. J. Breen (2010) | 10.1007/s00270-010-9954-3 | Conference abstract |
| G. Cappiello (2022) | 10.1109/iWAT54881.2022.9811044 | Not comparative study |
| K. Chang (2002) | NA | Case series, not comparative study |
| H. C. Chiu (2015) | 10.1111/bju.13057 | Conference abstract |
| M. Cody O'Dell (2015) | 10.1016/j.jvir.2015.03.003 | Letter |
| E. Collins (2017) | 10.1016/j.ejso.2017.10.007 | Conference abstract |
| N. M. Datrice (2011) | 10.1245/s10434-011-1552-3 | Conference abstract |
| J. J. Del Pizzo (2006) | 10.1007/s11934-006-0043-8 | Report |
| H. Fallahi (2017) | NA | Conference abstract |
| F. Fintelmann (2014) | 10.1016/j.jvir.2013.12.077 | Conference abstract |
| N. E. Frenk (2016) | 10.6061/clinics/2016(10)08 | Not comparative study |
| D. T. Ginat (2010) | 10.1053/j.tvir.2010.02.003 | Not comparative study |
| R. Giordano (2010) | 10.1530/eje-09-0957 | No interventional procedure |
| I. Haxhiu (2013) | 10.1016/S1569-9056(13)62029-1 | Conference abstract |
| R. Hodin (2014) | 10.1067/j.cpsurg.2014.01.001 | Not comparative study |
| H. Inoue (1997) | 10.2214/ajr.168.5.9129420 | Not comparative study |
| G. Argentesi (2022) | 10.1530/endoabs.86.P153 | Conference abstract |
| A. Khatun | 10.1186/ISRCTN11531672 | Ongoing clinical trial |
| M. Kato (2011) | 10.1089/end.2011.2003.supp | Conference abstract |
| A. N. Keeling (2009) | 10.1016/j.jvir.2009.03.039 | Letter |
| J. H. Kim (2022) | 10.3803/EnM.2022.1391 | Not comparative study |
| J. Lafemina (2012) | 10.1002/jso.23112 | Review |
| S. H. Lee (2017) | 10.1055/s-0042-118165 | Supplementary material |
| S. Y. Liu (2016) | 10.1148/radiol.2016152277 | Not comparative study |
| S. Y. Liu (2010) | 10.1097/SLA.0b013e318f66936 | Liu's comparative study from 2016 and 2010 includes duplicate cases. |
| C. H. Lo (2020) | 10.6515/acs.202003_36(2).20190812c | Not comparative study |
| J. Marescaux (2004) | 10.1001/jama.292.18.2214-c | Letter |
| L. A. McDuffie (2016) | 10.2217/ije-2015-0003 | Review |
| M. Mendiratta-Lala (2010) | 10.1148/radiol.10100690 | Not comparative study |
| R. Moll (2004) | 10.1055/s-2004-824849 | Not comparative study |
| M. Naruse (2017) | NA | Conference abstract |
| Z. Zhu | NA | Unpublished clinical trial |
| T. F. Nunes (2016) | 10.2214/AJR.16.16207 | Not comparative study |
| M. Pedziwiatr (2014) | 10.1155/2014/658483 | Laparoscopy only study |
| B. B. Pua (2012) | 10.1002/jso.23191 | Review |
| P. Raje (2021) | 10.1016/j.jss.2021.08.006 | Not comparative study |
| C. Ren (2016) | 10.3109/02656736.2016.1164905 | Not comparative study |
| B. A. Sacks (2017) | 10.1097/med.0000000000000329 | Review |
| C. Siegel (2005) | 10.1097/01.ju.0000171854.39368.f6 | Abstract |
| H. Soga (2009) | 10.1159/000200804 | Not comparative study, retrospectively analyzed 6 cases of adrenal cortical carcinoma |
| L. F. Starker (2016) | 10.1067/j.cpsurg.2016.04.002 | Not comparative study |
| O. Steichen (2016) | 10.1016/j.ando.2016.01.009 | Not comparative study |
| C. S. L. Tong (2011) | NA | Not comparative study |
| K. Takase | NA | Ongoing clinical trial |
| K. Takase | NA | Ongoing clinical trial |
| A. Vaidya (2017) | 10.4158/ep161717.Ra | Review |
| A. M. Venkatesan (2010) | 10.1053/j.tvir.2010.02.004 | Not comparative study |
| R. Viterbo (2005) | 10.1097/01.ju.0000165654.34635.ad | No interventional procedure |
| F. J. Wolf (2009) | NA | Review |
| M. H. Wu (2019) | 10.1097/mnm.0000000000000987 | Low number of radiofrequency ablation cases, insufficient data |
| K. Yamakado (2014) | 10.1055/s-0034-1373797 | Not comparative study |
| R. Yang (2014) | 10.1089/end.2013.0635 | A comparison of retroperitoneoscopic-guided cool-tip radiofrequency ablation and laparoscopic adrenalectomy. |
| M. Yatabe (2020) | 10.1038/s41440-020-0445-x | Focuses on adrenal vein sampling |
| H. Zhang (2020) | 10.1111/jch.13960 | Not comparative study |
| Z. Zhao (2021) | 10.1161/CIRCULATIONAHA.121.054318 | Compares adrenal ablation to medication therapy |
| J. Zuboy (2000) | NA | Abstract |
| J. Wood (2003) | 10.1002/cncr.11084 | Not comparative study |
| D. Szejnfeld (2015) | 10.1016/j.jvir.2015.06.019 | Not comparative study |
| Z. Liu (2020) | 10.5603/EP.a2019.0054 | Focuses on adrenal vein sampling, not comparative study |
| I. Hartmann (2014) | 10.5507/bp.2014.059 | Not comparative study |
| P. H. Graham (2016) | 10.1067/j.cpsurg.2016.04.002 | Not comparative study |

NA: not applicable

**Supplementary Table 4.** Eligibility criteria of each included article

| **Author (year)** | **Inclusion criteria** | **Exclusion criteria** |
| --- | --- | --- |
| Liu et al. (2016) | Age 18–80 years, aldosterone-producing adenoma (APA) < 4 cm. | Bilateral adrenal adenomas, multiple adrenal tumors, hormone co-secreting tumors, other concomitant adrenal diseases, potentially malignant tumors, heterogeneous contrast enhancement pattern, absolute contrast washout<60 per cent on delayed imaging, evidence of major vascular or tissue invasion, inaccessible APA location for percutaneous treatment, refusal to undergo laparoscopic adrenalectomy and radiofrequency ablation. |
| Yang et al. (2016) | Not defined | Not defined |
| Sarwar et al. (2016) | All patients referred for AVS at the AVS referral center were reviewed for study eligibility. AVS was performed using a standard technique. In patients with a selectivity index (SI = plasma cortisol in adrenal vein/plasma cortisol in inferior vena cava) greater than 2 in non-stimulated samples of each adrenal vein, a lateralization index (LI; [plasma aldosterone of dominant adrenal vein/plasma cortisol of dominant adrenal vein]/ [plasma aldosterone of non-dominant adrenal vein/plasma cortisol of non-dominant adrenal vein]) was calculated in post-ACTH samples. Unilateral PA was defined as patients with a LI > 4.0, per published guidelines. | Patients were excluded if adrenal veins were not successfully catheterized (SI <2), if the patient did not have an identifiable nodule on cross-sectional imaging, if the LI was less than 4, or if the patient was lost to follow-up. |
| Cano‑Valderrama et al. (2021) | PA was diagnosed when the patient had an ARR higher than 30 and at least one of the following: – A confirmatory 25 mg 2 h captopril test.  – Spontaneous hypokalemia, plasma renin below detection levels together with a serum aldosterone concentration higher than 200 pg/mL.  Unilateral PA was diagnosed when a lateralization index (LI) higher than four was found in adrenal venous sampling (AVS). AVS was only considered successful if the selectivity index (SI) (SI=cortisol in the adrenal vein/ cortisol in the inferior vena cava) was higher than two in both adrenal veins. Recently, they have also used the SI with androstenedione, which must be higher than three. Those patients with an unsuccessful AVS due to anatomical abnormalities or who did not undergo AVS were evaluated with SPECT/CT scintigraphy and/or CT. | Patients did not meet diagnostic criteria of primary aldosteronism, patients lost during the follow-up. |
| Sun et al. (2022) | Patients with APA were identified based on macroadenoma >1 cm on adrenal CT and enrolled in this trial. Patients were eligible for adrenal ablation if (1) they refused surgery and had a poor response to MRA treatment (patients could not endure the side effects of MRA or their blood pressure and biochemical parameters were not well improved, although they took a full dose of MRA) and (2) patients had a high surgical risk or were unsuitable for surgery after consulting with the urological surgeon | Patients were not considered for adrenal ablation therapy if they had (1) a history of serious contrast agent allergy; (2) severe cardiac, renal, and liver disease; (3) pregnancy or lactation; or (4) some serious comorbidities, such as mental diseases or malignant tumors. |

**Supplementary Table 5.** Baseline diagnostic and treatment characteristics of included studies

|  | | **Ablation** | | | | **Laparoscopic adrenalectomy** | | |
| --- | --- | --- | --- | --- | --- | --- | --- | --- |
| **Author (year)** | **Diagnostic method** | **Type of ablation, number of patients** | **Tumor size (mm)** | **Tumor laterality (right/left)** | **Number of patients** | | **Tumor size (mm)** | **Tumor laterality (right/left)** |
| Liu et al. (2016) | CT/MRI; laboratory test and AVS selectively | RFA; 36 | 16±5 | 17/19 | 27 | | 14±5 | 12/15 |
| Yang et al. (2016) | CT and laboratory test | RFA; 7 | 19 (11-25) | 2/5 | 18 | | 18 (8-25) | 8/10 |
| Sarwar et al. (2016) | AVS and laboratory test | RFA; 12 | 15.5± 5 | ND | 32 | | ND | ND |
| Cano‑  Valderrama et al. (2021) | Laboratory test, AVS + SPECT/CT selectively | RFA; 10 | ND | ND | 24 | | ND | ND |
| Sun et al. (2022) | AVS+CT | Ethanol; 52 | 12.2± 0.8 | 28/23 | 60 | | 15.4± 1.2 | 25/35 |

*** Parameters represented as mean with standard deviation, or median with range (minimum and maximum)**

CT: computed tomography; MRI: magnetic resonance imaging; AVS: adrenal vein sampling; SPECT: single-photon emission computed tomography; RFA: radiofrequency ablation; ND: not defined

**Supplementary Table 6.** Risk of bias assessment using the QUIPS tool

| **Author (year)** | **Study participation** | **Study attrition** | **Prognostic factor measurement** | **Outcome measurement** | **Study confounding** | **Statistical analysis and reporting** |
| --- | --- | --- | --- | --- | --- | --- |
| S. Y. Liu (2016) | Low | NA | Moderate | Low | Moderate | Low |
| Min‑Hsin Yang (2016) | Moderate | NA | Moderate | Moderate | Moderate | Low |
| Oscar Cano‑Valderrama (2021) | Moderate | NA | Moderate | Moderate | Moderate | Low |
| Ammar Sarwar (2016) | Low | NA | Low | Low | Moderate | Low |
| Fang Sun (2022) | Low | NA | Low | Moderate | Moderate | Low |

NA: not applicable

The included studies are mainly of medium risk in terms of risk of bias assessment.


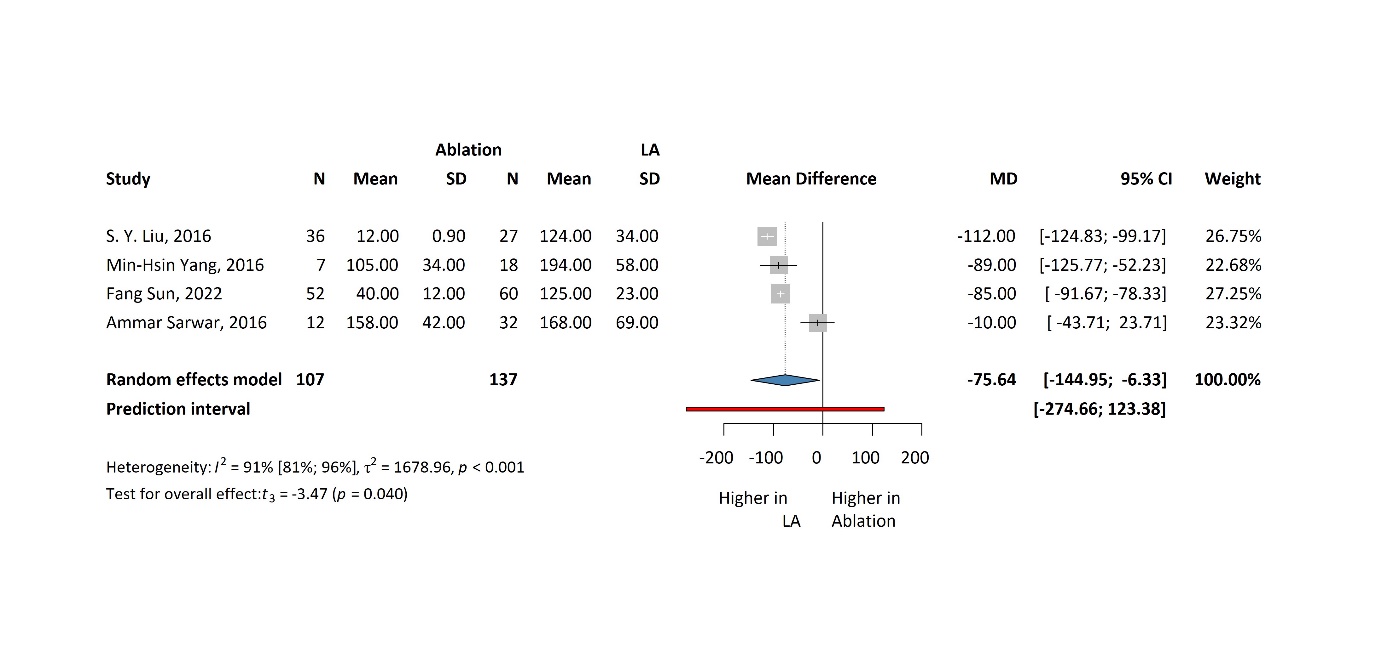
**Supplementary Figure 1.:** Summary forest plot of operation time (minutes)

**Supplementary Figure 2.:** Summary forest plot of length of hospital stay (days)


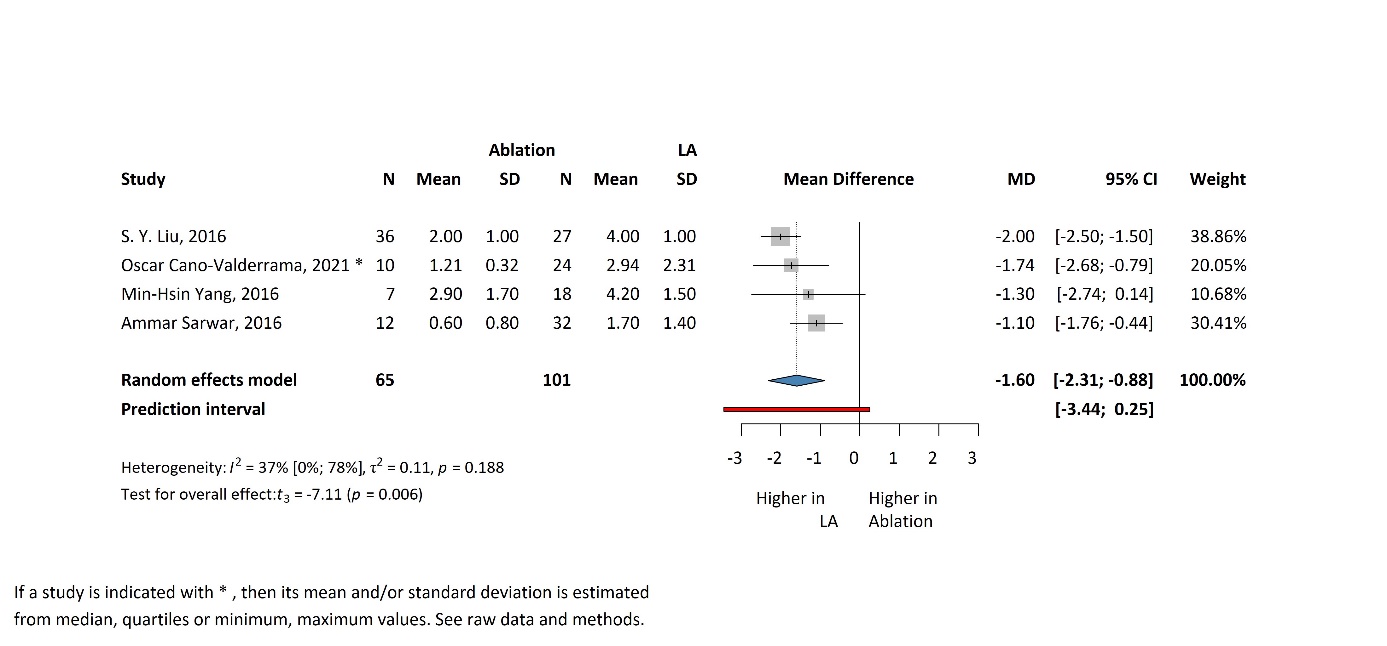


**Supplementary Figure 3.:** Summary forest plot of perioperative blood loss (ml)


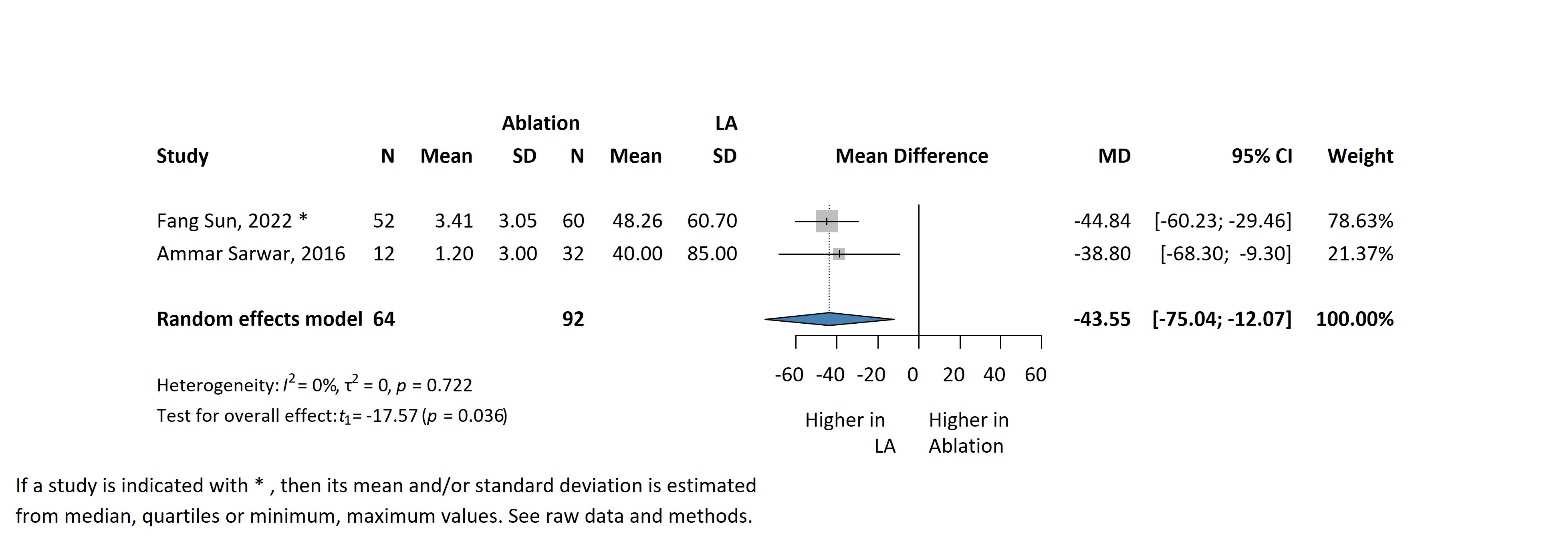


**Supplementary Figure 4.:** Summary forest plot of postoperative pain treatment


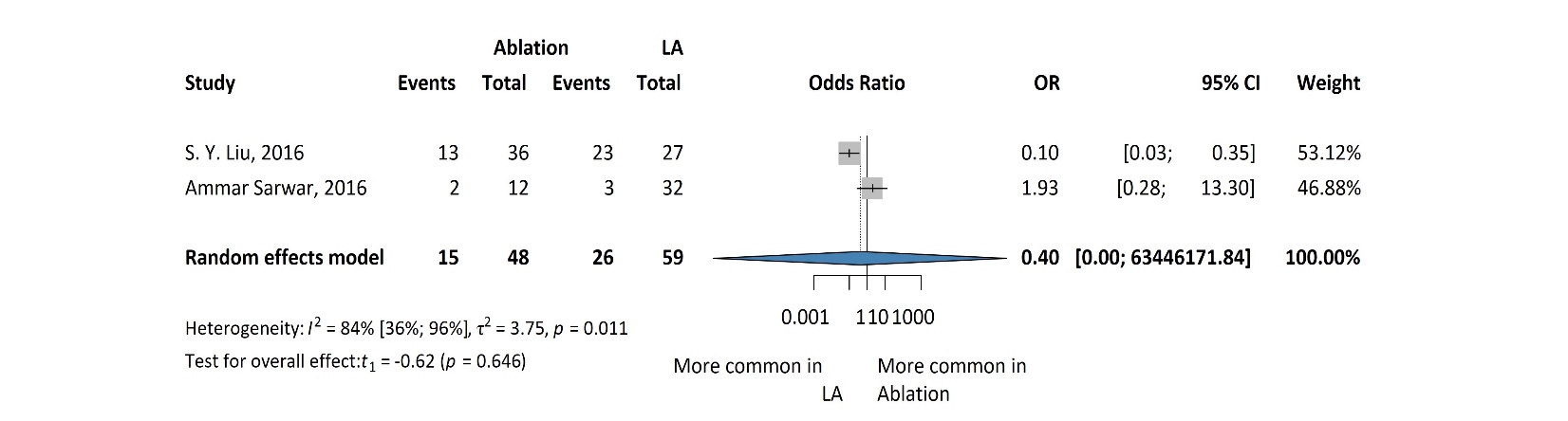

Supplement: Multimedia component 1 [file mmc1.docx]
